# Eliciting patient-important outcomes through group brainstorming: when is saturation reached?

**DOI:** 10.1186/s41687-019-0097-2

**Published:** 2019-02-04

**Authors:** Marianna LaNoue, Alexzandra Gentsch, Amy Cunningham, Geoffrey Mills, Amanda M. B. Doty, Judd E. Hollander, Brendan G. Carr, Larry Loebell, Gail Weingarten, Kristin L. Rising

**Affiliations:** 1 College of Population Health and Department of Family and Community Medicine, Thomas Jefferson University, 1015 Walnut St., suite 401, Philadelphia, PA 19107 USA; 2 Department of Emergency Medicine, Thomas Jefferson University, Philadelphia, USA; 3 Department of Family and Community Medicine, Thomas Jefferson University, Philadelphia, USA; 4 Voicing Outcomes Important for Care (VOICe) Study, Patient and Key Stakeholder Advisory Board (PAKSAB) member, Philadelphia, PA USA

## Abstract

**Purpose:**

Group brainstorming is a technique for the elicitation of patient input that has many potential uses, however no data demonstrate concept saturation. In this study we explore concept saturation in group brainstorming performed in a single session as compared to two or three sessions.

**Methods:**

Fifty-two predominately African American adults patients with moderately to poorly controlled Diabetes Mellitus participated in three separate group brainstorming sessions as part of a PCORI-funded group concept mapping study examining comparing methods for the elicitation of patient important outcomes (PIOs). Brainstorming was unstructured, in response to a prompt designed to elicit PIOs in diabetes care. We combined similar brainstormed responses from all three sessions into a ‘master list’ of unique PIOs, and then compared the proportion obtained at each individual session, as well as those obtained in combinations of 2 sessions, to the master list.

**Results:**

Twenty-four participants generated 85 responses in session A, 14 participants generated 63 in session B, and 14 participants generated 47 in session C. Compared to the master list, the individual sessions contributed 87%, 76%, and 63% of PIOs. Session B added 3 unique PIOs not present in session A, and session C added 2 PIOs not present in either A or B. No single session achieved >90% saturation of the master list, but all 3 combinations of 2 sessions achieved > 90%.

**Conclusions:**

Single sessions elicited only 63-87% of the patient-important outcomes obtained across all three sessions, however all combinations of two sessions elicited over 90% of the master list, suggesting that 2 sessions are sufficient for concept saturation.

**Trial registration:**

NCT02792777. Registered 2 June 2016.

Healthcare research is increasingly incorporating direct elicitation of patient input into activities including prioritization of research questions, development of patient-centered research protocols, and dissemination activities [1]. In addition, patient-reported outcome measures (PROMs) are now being tied to quality and reimbursement [2]. No matter the use case, patient input during the concept elicitation phase is vital to ensure high content validity of measures [3].

Group Concept Mapping (GCM) is a participatory research and engagement method used to engage research stakeholders for diverse purposes such as triangulating care priorities [4, 5] and including hard to reach stakeholder perspectives [6]. It has also been used for concept elicitation and conceptual domain organization in PROM development [2].

GCM involves engaging a group of individuals representing the population of interest to 1) *brainstorm* responses to a prompt designed for concept elicitation, 2) *organize* brainstormed ideas into conceptually-similar domains to produce a “‘concept map” of ideas and 3) *rat*e brainstormed responses along predefined dimensions. While interviews and focus groups are widely used approaches to engage patients for concept elicitation [1], they are time and resource intensive. GCM brainstorming offers an alternative method for concept elicitation that may be more efficient and comprehensive [7]. However, no empirical evidence exists to inform how many GCM group brainstorming sessions are needed for concept saturation.

Concept saturation in group-brainstorming is conceptually similar to thematic saturation in qualitative research. It refers to a determination that no new themes, codes or concepts are emerging, and data collection can end [8]. Although GCM literature uses the term saturation [9, 10], there are no published data regarding whether a single GCM brainstorming session is sufficient to achieve saturation of a conceptual domain. While researchers conducting a single group brainstorming activity could stop brainstorming and conclude saturation was reached when no new ideas emerged, this approach does not establish if saturation of the entire conceptual space has occurred.

Therefore, our aim in this study was to explore concept saturation across 3 sessions of group brainstorming in a population of patients with moderately to poorly controlled diabetes.

## Methods

This study is part of a larger Patient-Centered Outcomes Research Institute® (PCORI)-funded methodology project focused on comparing individual interviews with GCM brainstorming for their relative comprehensiveness and resource intensiveness (detailed methods published elsewhere) [7]. In the current study, we compared the outcomes elicited in a single GCM brainstorming session to those elicited across two and three sessions in order to establish when and whether concept saturation had occurred. All phases of the study were performed in collaboration with our Patient and Key Stakeholder Advisory Board (PAKSAB). The study received institutional IRB expedited approval.

### Participants & study setting

Participants were a convenience sample of 52 adults from a large Philadelphia health system. Potentially eligible participants were identified from the health system’s electronic medical record if they had an active diagnosis of type 1 or type 2 diabetes mellitus (DM); a primary care visit, emergency department (ED) visit, or hospital admission in the last 3 months; and moderately- to poorly-controlled DM. Moderate-to-poor control defined as at least two hemoglobin A1c measurements greater than 7.5 for the primary care setting, presentation to the ED with a DM-related problem, or admission to the hospital for a DM-related problem. We excluded patients if they had significant complications related to DM, were undergoing medical clearance, were in police custody or incarcerated, were non English-speaking, or had other major communication barriers.

Eligible individuals were contacted by phone and invited to participate for the upcoming GCM session, with targeted enrollment for a final show-rate of approximately 20 participants per session, consistent with sample sizes recommended in standard GCM guidance [11]. No-shows account for differences in the sample sizes at each session.

### Data collection

We performed in-person group brainstorming with three separate groups of participants (A, B, and C). Each brainstorming session lasted approximately 90 min. Written consent and a self-reported demographics survey were obtained from each participant. Participants were compensated $125 to complete all GCM activities.

Participants responded to a prompt developed by the research team and PAKSAB: *“You are here as a person with diabetes; when people with diabetes seek care, what are they hoping to improve or make happen?*” Participants were given notecards to record ideas before sharing them out loud. During brainstorming, all new ideas shared by the group were added to a list on a document projected at the front of the room. Brainstorming ended when participants had no new ideas to share in the group. Notecards were collected and unique written ideas were added to the list. Participants subsequently completed GCM sorting and rating; however, only the brainstorming data was analyzed to assess concept saturation.

### Data analysis

The research team and three PAKSAB members compiled a “master list” of patient-important outcomes (PIOs) by combining the lists of individual brainstormed ideas from the three groups, and then combining similarly themed ideas into PIO’s. For example, “get information on weight loss,” “understand how to control weight,” and “understand how to handle weight gain” were all merged into the PIO “understand how to control weight”. To minimize subjectivity in combining ideas, our entire PAKSAB made final decisions on merging ideas into PIOs, with disagreements reconciled by vote.

We compared the list of PIOs generated at each individual session and those obtained in each combination of 2 sessions to the master list to determine the proportion of final PIOs identified in each individual session or combination of sessions. We visualized unique and overlapping PIOs across all three sessions in a Venn diagram.

### Participant demographics

Participant demographics did not differ significantly on any measured variables across the three groups so we report the aggregated demographics. Participants had a mean age of 55.6 years (SD = 15, range 23–95), half (50%) were male, and they were predominately non-Hispanic (94%) African Americans (81%). Most (71%) had a high-school diploma or less, and more than half (56%) reported a household income of less than $50 K/year. Most (71%) reported a history of DM greater than 5 years, and all participants reported at least one other chronic health condition. They had a mean HbA1c of 9.2 (*SD* = 2.6), consistent with our recruitment strategy of moderately- to poorly-controlled DM patients. There were 24 participants in sessions A, and 14 participants each in the subsequent two sessions.

### Results: Outcomes elicitation

**Fig. 1:**
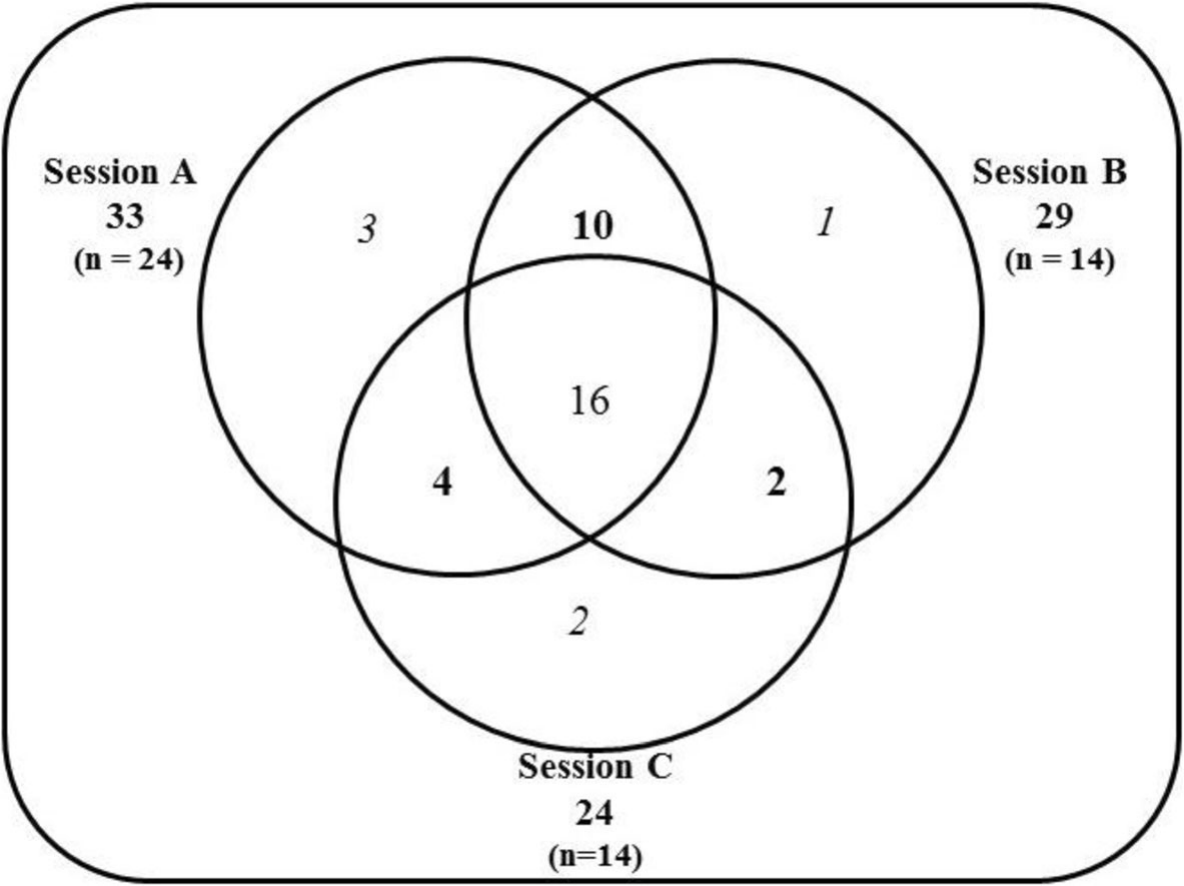
Venn Diagram of PIO Generation from the 3 Brainstorming Sessions. Legend: Bolded numbers in the outer circles indicate total PIOs identified in that session. The intersection of all 3 sessions shows 16 PIOs were common across the sessions. Italicized numbers in the outer circles show unique PIOs identified in that session, and bolded numbers in the inner circles show PIOs present in that 2-session combination

Participants generated 85 ideas in session A, 63 in session B, and 47 in session C. The per-person outcome generation in each session was 3.5, 4.8, and 3.3 respectively. After similar ideas were grouped into PIOs, the three sessions generated 38 unique PIOs. As displayed in Fig. 1, session A generated 87% of all PIOs (33/38). All combinations of 2 sessions produced over 90% of all PIOs. Sixteen PIOs were consistent across all 3 sessions. Session B added 3 unique PIOs not present in session A, and session C added 2 PIOs not present in either A or B.

## Discussion

We conducted group brainstorming with 3 groups of participants sampled from the same population to explore concept saturation. We stopped data collection within any single brainstorming session when our participants were unable to generate any new ideas. We found that single sessions elicited between 63 and 87% of the patient-important outcomes obtained across all three sessions, and that all combinations of two sessions elicited over 90% of the total list of PIOs.

As additional ideas emerged in each subsequent session, we acknowledge that true saturation was not reached. We suggest, however, that this level of saturation is sufficient for most applications. We performed this work as part of a larger study in which we also engaged 89 patients from the same population in individual interviews to explore the same conceptual space, thus allowing comparison of saturation between the two methods. When comparing results from GCM brainstorming and interviews, we found that any two GCM brainstorming sessions elicited more PIOs than the complete set of 89 interviews [7]. As the interview were conducted in a traditional qualitative framework where data was collected until thematic saturation was reached, this result supports the conclusion that 2 GCM brainstorming sessions are sufficient for concept elicitation in most cases. Individual researchers must balance the resources involved in generating new data with potentially diminishing returns in terms of conceptual sampling. There are likely some studies that require closer to 100% identification of all ideas, while 90% may be more than sufficient for others. Single sessions varied widely, with elicitation from 63 to 87%, suggesting that a single session may not be adequate for many studies.

There is recently increased attention to content and thematic saturation in the research literature, owing in part to regulatory changes allowing for PROMs to inform labelling claims for medical products [3]. Most of this increased attention is focused on strengthening inferences of saturation in the methods most commonly used in that setting: interviews and focus groups. Our study fills a gap in this literature by providing evidence of when concept saturation occurs in the group brainstorming setting as well.

### Limitations

We had a larger group and more brainstormed ideas in the first session, however, this group was not sufficient for concept saturation, and this group did not generate more PIOs per person. The finding that any combination of two groups was sufficient for saturation suggests that group size did not significantly affect results, as the two smaller groups in combination were still able to generate ≥90% of the PIOs. Also, the larger group generated at most two additional unique PIOs relative to the smaller groups, suggesting that group size has only a small effect on number of unique PIOs obtained. This work was performed by a single research team in a population of urban patients with moderately- to poorly controlled diabetes within one health system. Therefore, we do not know if a similar study in a different setting would produce different results in terms of the absolute number of ideas generated, or with respect to the saturation of the conceptual space in a single vs multiple sessions. We speculate that the homogeneity of our sample may have enabled us to reach saturation more quickly, as their range of experiences and preferences might be expected to be somewhat similar. This is a limitation inherent to any qualitative method used for concept elicitation, however, thus researchers should be mindful of how participants’ characteristics may affect the scope of the conceptual space.

We chose patients with diabetes mellitus (DM) as the population for this study because, despite the existence of evidence-based treatment guidelines, DM remains a poorly controlled condition for many patients. This suggests that there may be gaps in understanding and addressing patient priorities related to seeking care for diabetes. Our prompt was designed to elicit outcomes important to this specific group of patients and therefore intentionally does not sample the entire conceptual space of all outcomes potentially important to diabetes patients. It is possible that a group of patients sampled from a different population may produce more (or fewer) total outcomes; it is not possible in this data to infer how that may impact saturation of the conceptual space during group brainstorming. Further work should be done to explore these considerations. Despite these limitations, this study is the first to explore concept saturation in group brainstorming.

## Conclusions

Two group brainstorming sessions were sufficient to sample over 90% of the conceptual space obtained across all 3 sessions in a population of urban patients with moderately to poorly controlled diabetes. We conclude that when seeking to identify patient-important outcomes for research or clinical care, two group brainstorming sessions are sufficient to reach relative concept saturation while minimizing resource utilization.

## Abbreviations

DM: Diabetes mellitus
ED: Emergency Department
GCM: Group Concept Mapping
PAKSAB: Patient and Key Stakeholder Advisory Board
PCORI: Patient-Centered Outcomes Research Institute®
PIOs: Patient-important outcomes
PROMs: Patient-Reported Outcome Measures
